# Possible use of pulmonary artery stiffness in screening for portopulmonary hypertension

**DOI:** 10.1002/jcu.23240

**Published:** 2022-07-14

**Authors:** Jeroen N. Wessels, Frances S. de Man

**Affiliations:** ^1^ Department of Pulmonary Medicine Amsterdam UMC location Vrije Universiteit Amsterdam Amsterdam The Netherlands; ^2^ Amsterdam Cardiovascular Sciences Pulmonary Hypertension and Thrombosis Amsterdam The Netherlands

Portopulmonary hypertension (POPH) is characterized by portal hypertension leading to splanchnic vasodilatation and formation of portosystemic shunts. These phenomena contribute directly to a decrease in peripheral vascular resistance, which is exacerbated by vasoactive substances able to bypass the liver through the portosystemic shunts, ultimately leading to a hyperdynamic state.^1^ The increased blood flow itself may increase pulmonary artery pressure, also in the absence of pulmonary vascular remodeling. True POPH with increased pulmonary vascular resistance (PVR ≥3 Wood units) is found in approximately 5% of liver transplantation candidates.^2^ Although this prevalence is relatively low, it is important to screen for POPH through echocardiography as the 5‐year survival is only 40%.^3^ Screening is especially important in liver transplantation candidates as the mortality after liver transplantation is higher when pulmonary hypertension (PH) is present, but clear cutoffs for estimated pulmonary artery pressure are lacking.^4^ When systolic pulmonary artery pressure (sPAP) is >50 mmHg and/or signs of right ventricular (RV) hypertrophy or dysfunction are present, a diagnostic right heart catheterization is recommended. However, with lower values of estimated sPAP, it is not clear whether invasive catheterization is necessary as moderate elevation of sPAP may simply be a consequence of a hyperdynamic state. Therefore, a more detailed screening tool, using other non‐invasive parameters may improve screening for POPH (Figure 1).

**FIGURE 1:**
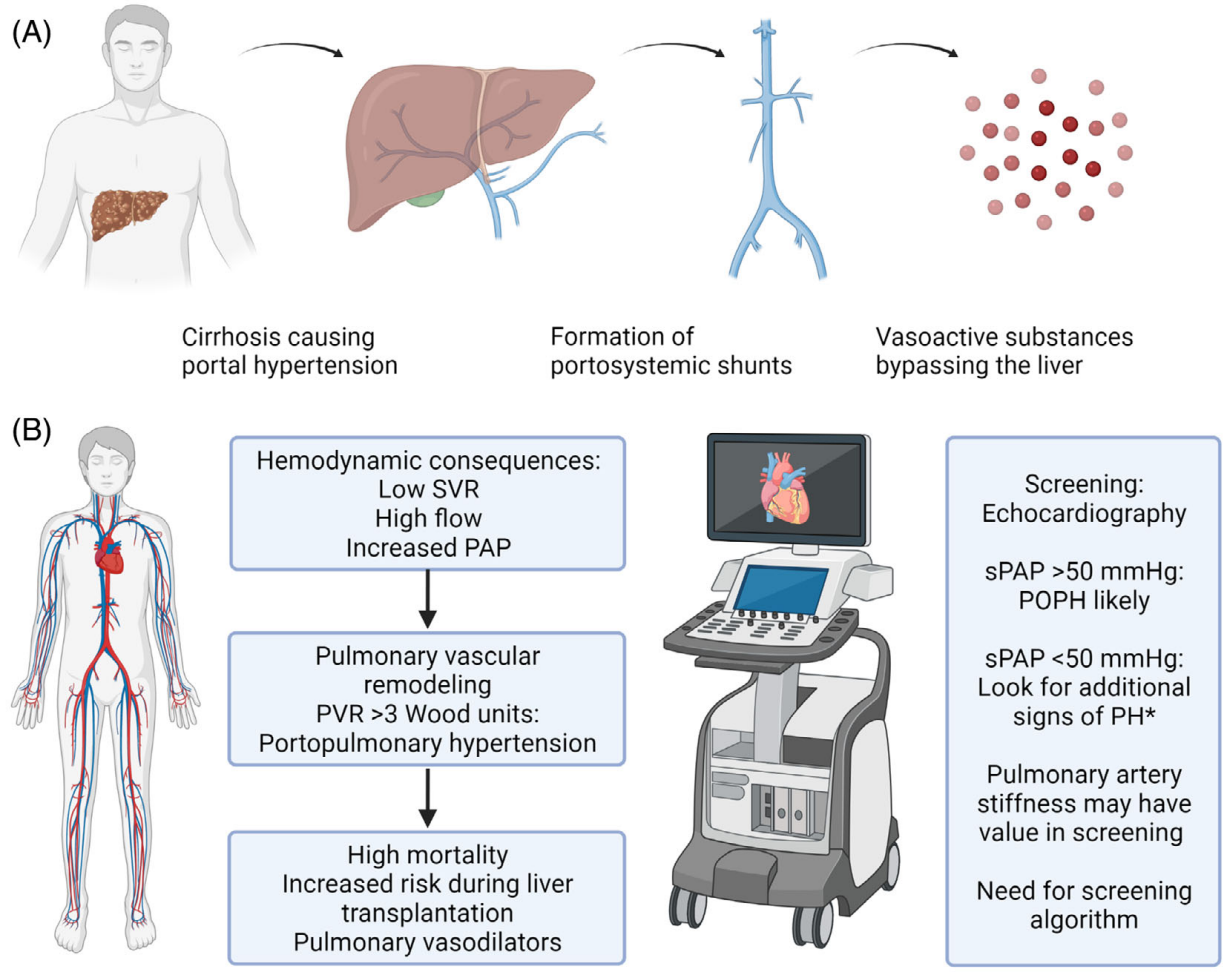
(A) Disease processes ultimately leading to portopulmonary hypertension. Patients with liver cirrhosis may develop portal hypertension, portosystemic shunts decrease systemic vascular resistance and vasoactive substances may bypass the liver through these shunts. (B) A hyperdynamic state with low SVR, high cardiac output and increased PAP may ensue. When pulmonary vascular remodeling is present, PVR increases, inducing true portopulmonary hypertension with increased mortality and risk during liver transplantation. Treatment with pulmonary vasodilators is important in these patients. Screening echocardiography is mainly based on estimation of sPAP, but in patients with sPAP <50 mmHg it is difficult to distinguish POPH from a hyperdynamic state. *RV/left ventricle basal diameter ratio >1, flattening of the interventricular septum, inferior cava diameter >21 mm with decreased inspiratory collapse, right atrial area (end‐systole) >18 cm^2^, RV outflow tract acceleration time <105 ms, early diastolic pulmonary regurgitation velocity >2.2 mm/s and pulmonary artery diameter >25 mm. SVR indicates systemic vascular resistance; PAP, pulmonary artery pressure; PVR, pulmonary vascular resistance; POPH, portopulmonary hypertension. Created with biorender.com

In this issue of the *Journal of Clinical Ultrasound* Elçioğlu et al evaluate whether echocardiographic measurement of pulmonary artery stiffness (PAS) is elevated in cirrhosis patients and whether it is correlated with RV function.^5^ Consecutive cirrhosis patients undergoing liver transplantation (*n* = 52) and age and sex matched healthy controls (*n* = 59) underwent transthoracic echocardiography. Besides conventional measurements, the PAS was determined by measuring pulmonary artery flow approximately 1 cm distal to the pulmonary valve from a parasternal short axis view and dividing the maximal frequency shift by pulmonary acceleration time. In cirrhosis patients, RV fractional area change was lower than in controls (45.31 ± 3.85% vs. 49.66 ± 3.62%), while tricuspid annular plane systolic excursion was not different. Both RV and right atrial diameter were higher in cirrhosis patients, suggesting that the right heart was volume‐overloaded, pressure‐overloaded or both. Estimated sPAP (27.69 ± 3.91 vs. 23.37 ± 3.81 mmHg) and PAS (20.52 ± 6.52 vs. 13.73 ± 2.05 kHz/s) were higher in patients and there was a weak correlation between these parameters. In addition, both showed weak correlations with RV functional parameters, especially RV fractional area change. None of the echocardiographic parameters were found to be correlated with the severity of cirrhosis (MELD or Child‐Pugh‐Turcotte scores).

Although the mean sPAP in these cirrhosis patients was well below the absolute cutoff of 50 mmHg to refer patients for catheterization,^4^ it was higher than in healthy controls. A sPAP between 35 and 50 mmHg must prompt the treating physician to look for additional signs of PH. In practice, there is considerable variation in screening and diagnosis of POPH between centers,^6^ while the guidelines give specific recommendations. According to the 2015 ESC/ERS guidelines on PH, screening includes an assessment of the ventricles, pulmonary artery and inferior vena cava and right atrium.^7^ Presence of one or more signs of PH raise the probability of PH and the guidelines recommend to consider catheterization in these patients. The following signs should be checked: RV/left ventricle basal diameter ratio >1, flattening of the interventricular septum, inferior cava diameter >21 mm with decreased inspiratory collapse and right atrial area (end‐systole) >18 cm^2^. With regards to the pulmonary artery, a measure of stiffness should be included (RV outflow tract acceleration time <105 ms), in addition to an early diastolic pulmonary regurgitation velocity >2.2 mm/s and pulmonary artery diameter >25 mm. PAS as evaluated in the study by Elçioğlu et al may provide additional information as it not only incorporates acceleration time, but also maximal flow velocity.^5^ Another reason it may have clinical relevance is that in group 1 PH patients (not POPH), PAS measured on cardiac magnetic resonance was associated with mortality.^8^ So far, no screening algorithm to calculate the risk of PH has been incorporated in the guidelines.^7^ As the proposed parameters and cutoffs have mostly been evaluated in different single‐center studies, it is difficult to compare their relative predictive value. A large, multicenter, diagnostic accuracy study comparing all relevant non‐invasive parameters including confirmation with catheterization may be necessary to create such an algorithm.

Some footnotes have to be placed regarding the current study. A relatively small, single center cohort was assessed and no information on outcome was reported. It would be of interest to study the relation between PAS and mortality or complications during or after liver transplantation. Also, the predictive value of PAS to distinguish between POPH, hyperdynamic state without elevation of PVR or no PH at all remains elusive. A cutoff for elevated PAS was not identified and the authors did not report whether patients with elevated PAS also had a sPAP above 35 mmHg. It is not possible to conclude from the descriptive data in this study whether increased PAS reflects early pulmonary vascular remodeling. Finally, there were only weak correlations between PAS and RV function and both may reflect a hyperdynamic state rather than pulmonary vascular remodeling.

To conclude, PAS appears to be of interest in the screening for POPH in liver transplantation candidates. Screening needs to be improved as POPH affects outcome after liver transplantation with mortality as high as 100% in patients with mean PAP >50 mmHg and as high as 50% when mean PAP is between 35 and 50 mmHg with PVR >3 Wood units.^9^ Furthermore, POPH patients have worse survival than other forms of group 1 PH and are undertreated.^3^ Timely initiation of medication (with phosphodiesterase 5 inhibitors, endothelin receptor antagonists, guanylate cyclase stimulators or prostacyclin analogs) may improve survival and serve as a bridge to liver transplant.^10^ Anecdotal reports suggest that pre‐treatment with the abovementioned drugs may improve the outcome after liver transplantation.^11^, ^12^ Finally, to make proper treatment decisions, a complete hemodynamic assessment remains necessary in all patients with high suspicion of POPH.^13^

## Data Availability

Data sharing is not applicable to this article as no new data were created or analyzed in this study.
